# Supplementary material for: A Likelihood Approach for Real-Time Calibration of Stochastic Compartmental Epidemic Models
Source: PLoS Comput Biol. 2017 Jan 17;13(1):e1005257. doi: 10.1371/journal.pcbi.1005257 (PMC5240920; doi:10.1371/journal.pcbi.1005257)
Supplement: S1 File — (TAR.GZ) [file pcbi.1005257.s014.tar.gz › HSPH_Online-SI-Revision/Additions/IPoi10/n100-extreme-SEITR_noisy/n100-extreme-SEITR_noisy_BENCH2_table4.pdf]

1	newly 3 weeks spec	1111.04	3672.76	{1814., 6027.}	230.569	0.471687	newly 3 weeks cumul	5404.68	13 690.4	{7243., 22 171.}	153.306	0.366283	newly 5 weeks spec	368.386	2285.68	{1041., 3942.}	520.457	0.733583	newly 5 weeks cumul	6347.06	18 887.9	{9707., 31 102.}	197.585	0.431505	newly 46 weeks cumul	9732.42	26 441.	{12 953., 43 675.}	171.679	0.384971
2	newly 3 weeks spec	909.	2930.4	{1274., 5552.}	222.494	0.42369	newly 3 weeks cumul	5060.	13 373.7	{6383., 24 960.}	164.613	0.356226	newly 5 weeks spec	300.	1269.29	{528., 2371.}	323.098	0.529266	newly 5 weeks cumul	5879.	16 585.9	{7731., 30 983.}	182.325	0.379183	newly 54 weeks cumul	6291.	18 885.9	{8644., 35 416.}	200.289	0.402221
3	newly 3 weeks spec	1244.	1090.28	{515., 2236.}	46.0168	0.226285	newly 3 weeks cumul	6664.	6151.42	{3007., 12 921.}	44.951	0.21094	newly 5 weeks spec	463.	360.42	{165., 734.}	46.5521	0.25236	newly 5 weeks cumul	7872.	7144.17	{3463., 15 000.}	45.1415	0.215051	newly 56 weeks cumul	8546.	7634.74	{3667., 16 092.}	45.2696	0.218788
4	newly 3 weeks spec	1162.	1884.46	{866., 3589.}	76.026	0.199856	newly 3 weeks cumul	6474.	9029.08	{4197., 16 954.}	61.02	0.184357	newly 5 weeks spec	412.	801.958	{357., 1494.}	100.427	0.234	newly 5 weeks cumul	7613.	11 055.	{5085., 20 704.}	64.3702	0.18668	newly 54 weeks cumul	8186.	12 481.3	{5728., 23 416.}	68.7059	0.190572
5	newly 3 weeks spec	334.	922.338	{431., 1711.}	176.871	0.368771	newly 3 weeks cumul	2304.	5508.97	{2520., 9994.}	140.518	0.315389	newly 5 weeks spec	78.	283.485	{127., 557.}	263.443	0.479628	newly 5 weeks cumul	2553.	6310.57	{2862., 11 469.}	148.361	0.327575	newly 56 weeks cumul	2676.	6659.74	{3007., 12 087.}	149.988	0.329747
6	newly 3 weeks spec	210.	1440.09	{623., 2281.}	585.757	0.737368	newly 3 weeks cumul	1688.	7666.18	{3390., 12 245.}	354.157	0.569736	newly 5 weeks spec	40.	507.732	{212., 778.}	1169.33	1.00091	newly 5 weeks cumul	1825.	9035.84	{3964., 14 348.}	395.114	0.605033	newly 55 weeks cumul	1849.	9769.55	{4285., 15 534.}	428.37	0.632451
7	newly 3 weeks spec	1990.	2271.82	{1206., 3614.}	41.4095	0.154315	newly 3 weeks cumul	8888.	8839.95	{4917., 14 162.}	34.2932	0.151361	newly 5 weeks spec	982.	1386.	{662., 2032.}	59.8811	0.171993	newly 5 weeks cumul	11 272.	12 007.9	{6518., 18 968.}	37.5403	0.151881	newly 47 weeks cumul	13 516.	16 325.4	{8497., 25 234.}	45.0332	0.154955
8	newly 3 weeks spec	1262.	2337.12	{1336., 3838.}	86.8356	0.222897	newly 3 weeks cumul	6258.	10 916.6	{6627., 18 061.}	76.258	0.211285	newly 5 weeks spec	510.	999.94	{561., 1608.}	97.8695	0.237318	newly 5 weeks cumul	7632.	13 460.9	{8068., 22 225.}	78.2589	0.212479	newly 54 weeks cumul	8594.	15 269.8	{9105., 25 223.}	79.625	0.213305
9	newly 3 weeks spec	351.	901.788	{384., 1611.}	157.472	0.334934	newly 3 weeks cumul	2373.	5398.44	{2525., 9709.}	128.854	0.290516	newly 5 weeks spec	96.	280.106	{131., 488.}	192.188	0.385921	newly 5 weeks cumul	2626.	6177.8	{2866., 11 084.}	136.279	0.302624	newly 56 weeks cumul	2718.	6518.91	{3039., 11 691.}	140.711	0.309904
10	newly 3 weeks spec	2417.	1350.21	{601., 2352.}	54.9892	0.356939	newly 3 weeks cumul	10 500.	6208.51	{2832., 10 659.}	52.0766	0.327933	newly 5 weeks spec	1167.	608.059	{262., 1048.}	57.6214	0.385893	newly 5 weeks cumul	13 414.	7728.32	{3515., 13 276.}	53.162	0.339218	newly 52 weeks cumul	16 196.	8965.5	{3992., 15 498.}	54.6522	0.355814
11	newly 3 weeks spec	2013.	2565.12	{1384., 3866.}	44.3257	0.156693	newly 3 weeks cumul	9231.	10 956.	{5925., 16 540.}	37.8374	0.142875	newly 5 weeks spec	1003.	1289.45	{690., 1948.}	45.6093	0.158985	newly 5 weeks cumul	11 702.	14 075.9	{7575., 21 261.}	39.1333	0.145765	newly 51 weeks cumul	13 920.	17 127.6	{9062., 25 839.}	41.0901	0.149838
12	newly 3 weeks spec	1233.	2680.83	{1314., 4841.}	119.38	0.28693	newly 3 weeks cumul	6317.	11 961.3	{6120., 21 095.}	92.8821	0.241659	newly 5 weeks spec	542.	1250.87	{594., 2299.}	132.71	0.306895	newly 5 weeks cumul	7681.	15 054.8	{7608., 26 743.}	99.031	0.252059	newly 52 weeks cumul	8581.	17 587.9	{8769., 31 470.}	107.448	0.266846
13	newly 3 weeks spec	1573.	1746.68	{824., 3235.}	45.2246	0.173261	newly 3 weeks cumul	7548.	8138.43	{4056., 14 731.}	42.4505	0.168138	newly 5 weeks spec	706.	776.676	{360., 1453.}	46.4423	0.179118	newly 5 weeks cumul	9330.	10 081.8	{4988., 18 291.}	43.0417	0.169993	newly 53 weeks cumul	10 733.	11 587.6	{5758., 21 166.}	43.4309	0.171447
14	newly 3 weeks spec	2628.	2345.02	{1090., 3613.}	42.634	0.202036	newly 3 weeks cumul	11 099.	8877.77	{4234., 14 200.}	39.4071	0.208325	newly 5 weeks spec	1313.	1450.13	{640., 2083.}	49.9072	0.185706	newly 5 weeks cumul	14 282.	12 192.3	{5683., 19 207.}	40.6824	0.202083	newly 46 weeks cumul	17 443.	16 996.9	{7610., 26 112.}	43.3091	0.187228
15	newly 3 weeks spec	1421.	2878.06	{1363., 4890.}	104.633	0.256649	newly 3 weeks cumul	6948.	10 862.2	{5584., 18 512.}	62.3777	0.180332	newly 5 weeks spec	621.	1803.34	{864., 2948.}	190.393	0.380329	newly 5 weeks cumul	8459.	14 960.5	{7482., 25 448.}	80.7434	0.21488	newly 47 weeks cumul	9505.	20 871.8	{9875., 34 740.}	120.67	0.282525
16	newly 3 weeks spec	1685.	2261.68	{1119., 4921.}	53.5815	0.175566	newly 3 weeks cumul	8356.	9414.17	{4883., 19 551.}	40.998	0.159344	newly 5 weeks spec	644.	1201.37	{570., 2675.}	91.8965	0.238278	newly 5 weeks cumul	10 017.	12 270.6	{6272., 25 939.}	46.1201	0.165062	newly 50 weeks cumul	11 020.	15 356.2	{7756., 32 791.}	56.118	0.179024
17	newly 3 weeks spec	244.	1538.28	{713., 2378.}	530.441	0.73464	newly 3 weeks cumul	2213.	8514.74	{4082., 13 258.}	284.76	0.526211	newly 5 weeks spec	41.	513.292	{236., 790.}	1151.93	1.02889	newly 5 weeks cumul	2358.	9922.57	{4748., 15 483.}	320.804	0.563914	newly 56 weeks cumul	2381.	10 616.3	{5088., 16 506.}	345.876	0.588587
18	newly 3 weeks spec	946.	1536.91	{784., 2674.}	69.8265	0.202022	newly 3 weeks cumul	4981.	8186.18	{4364., 13 599.}	70.006	0.202167	newly 5 weeks spec	314.	553.824	{275., 912.}	82.2405	0.227296	newly 5 weeks cumul	5835.	9658.84	{5151., 16 092.}	71.1374	0.204357	newly 55 weeks cumul	6316.	10 457.4	{5560., 17 393.}	71.1899	0.204273
19	newly 3 weeks spec	2246.	3711.5	{2066., 5834.}	68.9434	0.202723	newly 3 weeks cumul	9933.	14 977.6	{8804., 22 869.}	55.9695	0.175808	newly 5 weeks spec	1136.	2009.86	{1110., 3285.}	79.9337	0.224019	newly 5 weeks cumul	12 584.	19 721.8	{11 470., 30 624.}	61.2023	0.186936	newly 50 weeks cumul	14 784.	24 921.8	{14 283., 39 159.}	71.5203	0.208862
20	newly 3 weeks spec	688.	1796.03	{871., 2630.}	161.051	0.339036	newly 3 weeks cumul	3837.	9102.38	{4847., 14 074.}	137.423	0.320405	newly 5 weeks spec	219.	675.8	{302., 931.}	208.584	0.395614	newly 5 weeks cumul	4426.	10 895.6	{5653., 16 602.}	146.246	0.330689	newly 55 weeks cumul	4693.	11 924.	{6077., 18 101.}	154.092	0.341317
21	newly 3 weeks spec	566.	1955.72	{799., 3337.}	245.593	0.420046	newly 3 weeks cumul	3401.	10 333.4	{4622., 18 031.}	204.156	0.391306	newly 5 weeks spec	156.	677.789	{256., 1156.}	334.48	0.507061	newly 5 weeks cumul	3828.	12 163.7	{5390., 20 986.}	217.969	0.405371	newly 56 weeks cumul	3988.	13 081.	{5723., 22 513.}	228.155	0.416426
22	newly 3 weeks spec	1150.	1636.54	{634., 3061.}	72.7487	0.21632	newly 3 weeks cumul	5834.	7348.83	{3161., 13 570.}	62.8923	0.214487	newly 5 weeks spec	400.	753.441	{289., 1355.}	101.394	0.231179	newly 5 weeks cumul	6931.	9204.8	{3923., 16 937.}	66.4663	0.213727	newly 52 weeks cumul	7573.	10 719.8	{4499., 19 730.}	71.068	0.213447
23	newly 3 weeks spec	361.	1606.56	{687., 3201.}	345.03	0.585462	newly 3 weeks cumul	2348.	7804.29	{3394., 15 464.}	232.38	0.462332	newly 5 weeks spec	79.	672.22	{298., 1292.}	750.912	0.86576	newly 5 weeks cumul	2588.	9518.73	{4101., 18 785.}	267.803	0.505376	newly 53 weeks cumul	2670.	10 725.3	{4574., 21 204.}	301.696	0.543028
24	newly 3 weeks spec	617.	1997.28	{806., 3713.}	223.709	0.417264	newly 3 weeks cumul	3939.	10 219.2	{4479., 18 921.}	159.846	0.339245	newly 5 weeks spec	171.	737.317	{267., 1333.}	331.179	0.53271	newly 5 weeks cumul	4404.	12 173.4	{5222., 22 383.}	176.418	0.361552	newly 55 weeks cumul	4602.	13 256.4	{5649., 24 247.}	188.058	0.377572
25	newly 3 weeks spec	1330.	3285.77	{1815., 5054.}	147.05	0.351969	newly 3 weeks cumul	7007.	13 717.6	{7875., 21 045.}	95.956	0.259331	newly 5 weeks spec	443.	1693.84	{884., 2596.}	282.356	0.534048	newly 5 weeks cumul	8227.	17 790.	{9991., 27 373.}	116.239	0.298909	newly 51 weeks cumul	8870.	21 824.2	{12 056., 33 757.}	146.045	0.352504
26	newly 3 weeks spec	920.	2989.57	{1517., 5160.}	224.953	0.458703	newly 3 weeks cumul	5136.	12 033.1	{6464., 20 768.}	134.29	0.328328	newly 5 weeks spec	345.	1643.72	{788., 2894.}	376.44	0.61161	newly 5 weeks cumul	6057.	15 902.3	{8370., 27 500.}	162.544	0.372997	newly 49 weeks cumul	6590.	20 212.5	{10 305., 34 800.}	206.715	0.43473
27	newly 3 weeks spec	2118.	1426.25	{734., 2542.}	43.156	0.258376	newly 3 weeks cumul	9250.	6057.15	{3130., 10 774.}	43.4683	0.263975	newly 5 weeks spec	1079.	745.119	{368., 1380.}	43.0664	0.254666	newly 5 weeks cumul	11 803.	7839.36	{4041., 14 054.}	43.2802	0.260986	newly 49 weeks cumul	14 480.	9744.69	{4995., 17 430.}	43.1644	0.258672
28	newly 3 weeks spec	196.	984.265	{468., 1815.}	402.176	0.637825	newly 3 weeks cumul	1632.	5573.19	{2673., 10 209.}	241.495	0.474401	newly 5 weeks spec	32.	328.971	{142., 586.}	928.033	0.948045	newly 5 weeks cumul	1750.	6471.32	{3100., 11 819.}	269.79	0.508568	newly 56 weeks cumul	1781.	6912.15	{3287., 12 667.}	288.105	0.529476
29	newly 3 weeks spec	1912.	1257.01	{560., 1985.}	40.9136	0.256484	newly 3 weeks cumul	9043.	5988.36	{2702., 9254.}	40.0499	0.250488	newly 5 weeks spec	849.	557.209	{241., 865.}	40.9367	0.259128	newly 5 weeks cumul	11 162.	7383.61	{3281., 11 401.}	40.1918	0.25164	newly 53 weeks cumul	12 786.	8445.67	{3778., 13 048.}	40.348	0.252689
30	newly 3 weeks spec	1221.	1629.13	{764., 2646.}	57.0535	0.181389	newly 3 weeks cumul	6812.	9012.87	{4541., 14 651.}	54.1588	0.174472	newly 5 weeks spec	428.	552.279	{273., 892.}	56.2701	0.185175	newly 5 weeks cumul	7969.	10 513.4	{5336., 17 106.}	54.3654	0.175377	newly 56 weeks cumul	8592.	11 251.5	{5720., 18 344.}	54.0938	0.175595
31	newly 3 weeks spec	1586.	2651.6	{1514., 3948.}	70.6063	0.194986	newly 3 weeks cumul	7252.	10 141.6	{6124., 15 395.}	48.8519	0.153645	newly 5 weeks spec	689.	1592.37	{892., 2356.}	131.372	0.310988	newly 5 weeks cumul	9024.	13 786.7	{8151., 20 861.}	58.4097	0.171424	newly 46 weeks cumul	10 290.	18 935.7	{10 795., 28 465.}	85.3948	0.226094
32	newly 3 weeks spec	2184.	1623.91	{868., 2586.}	42.5851	0.232714	newly 3 weeks cumul	9583.	7471.51	{3997., 11 885.}	39.9985	0.211571	newly 5 weeks spec	1094.	740.493	{378., 1173.}	45.3382	0.264235	newly 5 weeks cumul	12 124.	9321.81	{4937., 14 800.}	40.8024	0.217913	newly 52 weeks cumul	14 626.	10 824.3	{5694., 17 184.}	41.948	0.230032
33	newly 3 weeks spec	643.	328.787	{134., 616.}	63.7713	0.444075	newly 3 weeks cumul	3804.	1990.56	{827., 3848.}	62.9691	0.432382	newly 5 weeks spec	197.	98.7213	{35., 193.}	64.4004	0.461552	newly 5 weeks cumul	4382.	2270.51	{942., 4385.}	63.2053	0.436626	newly 56 weeks cumul	4693.	2396.03	{991., 4631.}	63.4021	0.441448
34	newly 3 weeks spec	883.	1781.	{884., 4014.}	104.265	0.237607	newly 3 weeks cumul	4731.	7994.91	{3952., 17 641.}	76.2459	0.191369	newly 5 weeks spec	278.	828.91	{382., 1877.}	198.309	0.389996	newly 5 weeks cumul	5501.	10 041.7	{4894., 22 303.}	86.9039	0.208388	newly 52 weeks cumul	5915.	11 745.	{5684., 26 227.}	100.907	0.233184
35	newly 3 weeks spec	239.	1406.91	{767., 2308.}	488.664	0.734572	newly 3 weeks cumul	1921.	7882.16	{4373., 12 848.}	310.315	0.579928	newly 5 weeks spec	50.	468.253	{244., 770.}	836.507	0.934848	newly 5 weeks cumul	2064.	9159.	{5100., 14 922.}	343.75	0.613479	newly 56 weeks cumul	2096.	9791.39	{5460., 16 030.}	367.146	0.635422
36	newly 3 weeks spec	2432.	3542.44	{2058., 5131.}	53.3052	0.155523	newly 3 weeks cumul	10 891.	14 674.4	{9029., 21 397.}	43.0709	0.138955	newly 5 weeks spec	1211.	1832.6	{954., 2562.}	60.7653	0.162321	newly 5 weeks cumul	13 782.	19 071.1	{11 480., 27 562.}	46.6856	0.144902	newly 52 weeks cumul	16 479.	23 338.6	{13 641., 33 539.}	50.108	0.149748
37	newly 3 weeks spec	1023.	2329.87	{1265., 3845.}	127.804	0.309542	newly 3 weeks cumul	5532.	10 734.	{6067., 17 080.}	94.3471	0.247298	newly 5 weeks spec	383.	1034.24	{542., 1694.}	170.036	0.378457	newly 5 weeks cumul	6497.	13 320.	{7397., 21 280.}	105.189	0.268869	newly 53 weeks cumul	7036.	15 357.1	{8520., 24 561.}	118.277	0.294221
38	newly 3 weeks spec	915.	1967.2	{983., 3374.}	116.991	0.274855	newly 3 weeks cumul	5124.	8416.52	{4454., 14 495.}	71.6633	0.193033	newly 5 weeks spec	323.	990.377	{477., 1682.}	206.618	0.411591	newly 5 weeks cumul	5962.	10 801.9	{5630., 18 517.}	85.3162	0.216261	newly 50 weeks cumul	6391.	13 159.7	{6740., 22 641.}	108.031	0.258971
39	newly 3 weeks spec	1339.	2000.89	{941., 2906.}	64.6151	0.179916	newly 3 weeks cumul	6703.	8669.44	{4592., 13 091.}	48.8504	0.162926	newly 5 weeks spec	464.	1057.18	{423., 1342.}	132.766	0.248109	newly 5 weeks cumul	7906.	11 180.2	{5640., 16 332.}	57.7833	0.17271	newly 52 weeks cumul	8666.	13 530.1	{6475., 19 087.}	69.3753	0.183044
40	newly 3 weeks spec	1822.	645.213	{343., 1241.}	64.5876	0.497109	newly 3 weeks cumul	8114.	3205.33	{1686., 6157.}	60.4963	0.448169	newly 5 weeks spec	874.	264.053	{134., 507.}	69.7879	0.567579	newly 5 weeks cumul	10 273.	3885.99	{2039., 7463.}	62.1728	0.467286	newly 53 weeks cumul	12 291.	4358.36	{2279., 8409.}	64.5402	0.495625
41	newly 3 weeks spec	362.	1894.98	{788., 2470.}	423.476	0.604228	newly 3 weeks cumul	2774.	9727.35	{4465., 13 704.}	250.661	0.46477	newly 5 weeks spec	74.	669.95	{243., 813.}	805.338	0.8204	newly 5 weeks cumul	2996.	11 520.8	{5123., 15 900.}	284.54	0.497638	newly 56 weeks cumul	3059.	12 421.1	{5480., 17 029.}	306.051	0.517695
42	newly 3 weeks spec	2612.	2404.92	{1259., 4398.}	39.5288	0.181696	newly 3 weeks cumul	10 679.	9490.4	{5255., 17 117.}	37.3332	0.178472	newly 5 weeks spec	1374.	1390.91	{732., 2669.}	42.0255	0.173744	newly 5 weeks cumul	13 825.	12 715.5	{6973., 23 283.}	38.1293	0.176016	newly 47 weeks cumul	17 141.	16 875.9	{9083., 31 061.}	39.4539	0.16977
43	newly 3 weeks spec	2253.	1189.1	{597., 1972.}	51.9608	0.351073	newly 3 weeks cumul	10 165.	5509.44	{2741., 9129.}	50.5087	0.336101	newly 5 weeks spec	1018.	540.145	{256., 925.}	52.1215	0.352761	newly 5 weeks cumul	12 779.	6850.87	{3351., 11 445.}	51.	0.341355	newly 52 weeks cumul	15 131.	7939.82	{3838., 13 320.}	51.7382	0.349731
44	newly 3 weeks spec	1384.	2722.59	{1411., 5481.}	99.0408	0.251673	newly 3 weeks cumul	6755.	12 091.4	{6330., 23 286.}	82.3821	0.223253	newly 5 weeks spec	626.	1257.5	{620., 2607.}	103.611	0.255915	newly 5 weeks cumul	8295.	15 213.2	{7878., 29 660.}	86.5176	0.230051	newly 52 weeks cumul	9359.	17 799.7	{9202., 35 193.}	92.8115	0.241356
45	newly 3 weeks spec	1196.	2417.81	{1304., 3824.}	103.083	0.273249	newly 3 weeks cumul	6409.	11 598.2	{6309., 18 417.}	83.0411	0.234336	newly 5 weeks spec	536.	1016.84	{526., 1669.}	92.1327	0.251213	newly 5 weeks cumul	7757.	14 183.7	{7670., 22 547.}	84.8747	0.237707	newly 54 weeks cumul	8640.	16 005.1	{8582., 25 615.}	87.1582	0.242136
46	newly 3 weeks spec	1149.	531.353	{257., 892.}	57.3107	0.40631	newly 3 weeks cumul	6165.	3039.44	{1508., 4879.}	55.0589	0.379299	newly 5 weeks spec	377.	175.309	{87., 295.}	56.838	0.401815	newly 5 weeks cumul	7203.	3522.9	{1723., 5654.}	55.3497	0.382769	newly 56 weeks cumul	7768.	3754.5	{1838., 6032.}	55.7354	0.387502
47	newly 3 weeks spec	1358.	1810.15	{876., 2643.}	52.6709	0.159288	newly 3 weeks cumul	7212.	7857.1	{3908., 11 793.}	40.0874	0.151872	newly 5 weeks spec	550.	892.881	{416., 1335.}	72.5547	0.186723	newly 5 weeks cumul	8640.	10 022.3	{4878., 14 956.}	43.1648	0.15219	newly 51 weeks cumul	9536.	12 037.5	{5765., 17 901.}	48.4489	0.156408
48	newly 3 weeks spec	874.	1556.1	{809., 2406.}	82.1897	0.213715	newly 3 weeks cumul	5250.	7518.97	{4084., 11 823.}	54.4695	0.161555	newly 5 weeks spec	248.	655.471	{335., 1021.}	164.303	0.3585	newly 5 weeks cumul	5990.	9192.51	{4942., 14 449.}	61.8111	0.174699	newly 54 weeks cumul	6323.	10 355.7	{5516., 16 199.}	69.8419	0.190152
49	newly 3 weeks spec	879.	820.71	{383., 1278.}	40.3354	0.191407	newly 3 weeks cumul	4780.	3917.77	{1840., 5821.}	39.6221	0.210915	newly 5 weeks spec	321.	362.355	{178., 546.}	47.7138	0.187528	newly 5 weeks cumul	5641.	4829.58	{2266., 7084.}	39.4508	0.202633	newly 52 weeks cumul	6148.	5518.18	{2586., 8116.}	39.624	0.195328
50	newly 3 weeks spec	1513.	1518.84	{896., 2474.}	34.2331	0.146794	newly 3 weeks cumul	7746.	7190.88	{4283., 11 596.}	32.8331	0.152098	newly 5 weeks spec	636.	676.707	{395., 1108.}	35.7254	0.144916	newly 5 weeks cumul	9438.	8891.95	{5300., 14 395.}	33.0773	0.15115	newly 53 weeks cumul	10 556.	10 193.2	{6085., 16 592.}	33.364	0.148815
